# The Time Course Effects of Electroacupuncture on Promoting Skeletal Muscle Regeneration and Inhibiting Excessive Fibrosis after Contusion in Rabbits

**DOI:** 10.1155/2013/869398

**Published:** 2013-08-06

**Authors:** Rongguo Wang, Dan Luo, Cheng Xiao, Peng Lin, Shouyao Liu, Qianwei Xu, Yunting Wang

**Affiliations:** ^1^Trauma Department of Orthopedics, China-Japan Friendship Hospital, Beijing 100029, China; ^2^College of Acupuncture-Moxibustion and Tuina, Beijing University of Chinese Medicine, Beijing 100029, China; ^3^Institute of Clinical Medicine, China-Japan Friendship Hospital, Beijing 100029, China

## Abstract

The aim of this study was to investigate the longitudinal effects of electroacupuncture (EA) on Zusanli (ST36) and Ashi acupoints in promoting skeletal muscle regeneration and inhibiting excessive fibrosis after contusion in rabbits. Sixty rabbits were randomly divided into four groups: normal, contusion, EA, and recombinant human insulin-like growth factor-I (rhIGF-I). An acute skeletal muscle contusion was produced on the right gastrocnemius (GM) by an instrument-based drop-mass technique. EA was performed for 15 minutes every two days with 0.4 mA (2 Hz), and GM injections were executed with rhIGF-I (0.25 mL once a week). Rabbits treated with EA had a higher T-SOD and T-AOC serum activities and lower MDA serum level, the blood perfusion of which was also significantly higher. In the EA group, the diameter of the myofibril was uniform and the arrangement was regular, contrary to the contusion group. The number and diameter of regenerative myofibers and MHC expression were increased in the EA group. EA treatment significantly decreased fibrosis formation and reduced both GDF-8 and p-Smad2/3 expressions in injured muscle. Our data indicate that EA may promote myofiber regeneration and reduce excessive fibrosis by improving blood flow and antioxidant capacities. Additionally, EA may regulate signaling factor expression after contusion.

## 1. Introduction

Skeletal muscle injuries are very common, particularly in sports. Simultaneously, muscle strains or contusions cause the majority of such damage. After muscle injuries, a healing process that involves degeneration, inflammation, regeneration, and fibrosis is initiated. Although the process of muscle regeneration is activated shortly after injury, healing can be hindered by the development of fibrosis and affects the degree of recovery. Furthermore, the development of fibrosis predisposes the muscle to further injury [1]. Additionally, incomplete functional recovery also often occurs because of fibrosis after muscle contusions [2].

Currently, treatments with therapeutic ultrasound [3–5], low-energy laser [6, 7], and cell transplantation [8–10] are new approaches for promoting skeletal muscle regeneration. However, these treatments cannot reduce excessive fibrosis according to animal experiments and clinical research. Growth factors, such as basic fibroblast growth factor (bFGF), insulin-like growth factor-I (IGF-I), and nerve growth factor (NGF), improve muscle healing, but the postcontusion healing process remains incomplete [11]. If antifibrosis agents could prevent myofibroblast formation and improve muscle healing (while at the same time antagonizing transforming growth of factor-beta 1 (TGF-*β*1)), gene therapy and tissue engineering may still be required [11, 12]. Therefore, this strategy will be complex in the clinic. Similarly, although studies also demonstrated that other medications including relaxin, decorin, and losartan can efficiently prevent fibrosis and enhance muscle regeneration [13–15], further research is required before they can be used in the clinic. Furthermore, the costs and side effects of drugs must be considered.

Complementary medicine, such as acupuncture, has been extensively used in China and other countries. It has unique clinical effects for the treatment of numerous diseases such as gouty arthritis [16], ankle sprain [17], and posttraumatic stress disorder [18]. One of the methods of acupuncture, electroacupuncture (EA), is based on traditional acupuncture but is aided by modern technology. EA shows a better curative effect and a wider range of applications than traditional acupuncture in some aspects [19, 20]. Recently, studies reported that acupuncture has positive influences on wound healing [21, 22] and functional peripheral nerve regeneration [23]. Using mRNA fingerprinting and bioinformatics approaches, Takaoka et al. [24] revealed that EA could promote cell proliferation in skeletal muscles leading to muscle repair. Our preliminary study has also demonstrated that EA not only promoted skeletal muscle regeneration but also reduced excessive fibrosis [25]. It also must be noted that the exact mechanism of EA action for both of these effects is poorly understood. 

Therefore, we conducted the following experimental research to determine the mechanism of action. By performing EA on Zusanli and Ashi acupoints, we examined the repair process of acute contusion in rabbits. We observed effects of EA on myofiber regeneration, ultrastructure of injured gastrocnemius (GM), local microcirculation perfusion, oxidative stress levels, collagen deposition, and changes in myosin heavy chain (MHC), growth differentiation factor-8 (GDF-8), and p-Smad2/3. The results obtained provide certain reliable experimental evidence for explaining the effect of EA on the repair of skeletal muscle injury after contusion.

## 2. Materials and Methods

### 2.1. Animal Model and Experimental Groups

**Figure 1:**
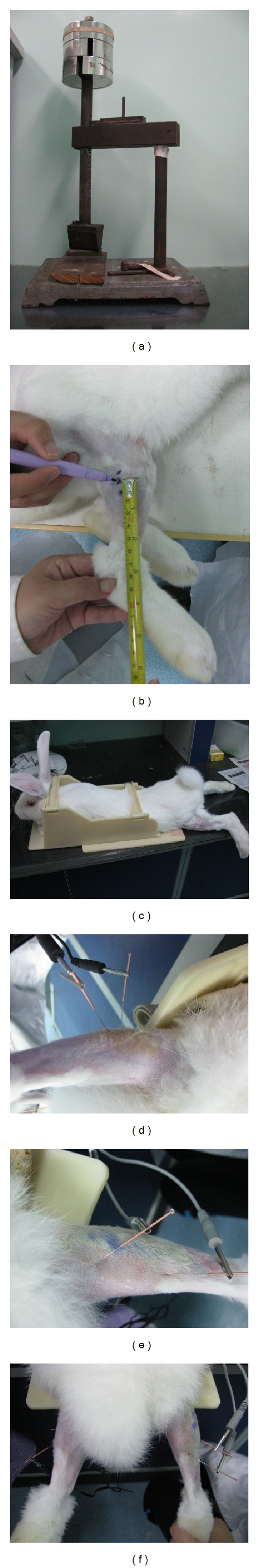
An acute skeletal muscle contusion model was established (a-b). EA treatment after contusion (c–f). (a) The contusion device. (b) The hit position: extending the knee and dorsi-flexing the ankle to 90°. The GM was struck 80 mm away from the rear edge of the calcaneus. The hit kinetic energy was calculated as follows: 7.5 kg × 0.13 m × 9.8 N/kg = 9.555 J. (c) The fixed position of rabbits when starting EA. (d) EA at the normal side ST36: the main needle was inserted into acupoint areas, and the auxiliary needle was placed 5 mm away from the main needle. (e) EA at Ashi acupoints of the injured side, which were located 10 mm from the proximal end (as anode) or the distal end (as cathode) at the contusion midpoint. (f) EA at ST36 and Ashi acupoints.

New Zealand rabbits (either male or female, weight: 2.0 ± 0.2 kg) were administered intravenous anesthesia through the marginal vein of the ear with 3% pentobarbital sodium (30 mg/kg of body weight). The animals were positioned with their right side fixed to the experiment table. The animal's hind limb was positioned by extending the knee and dorsiflexing the ankle to 90° to display the GM. After anesthetizing the animals, blunt injuries were inflicted on the GM at a location 80 mm from the rear edge of the calcaneus with a crushing machine using a drop-mass technique. The injury area was 1 cm^2^ and had an energy of 9.555 J [26–28] (Figures 1(a)-1(b)). Before the animals returned to normal activities, we confirmed that the skin was intact and there were no tibia or fibula fractures. Although the anatomy, pathology, and function tests confirmed injury to the GM accompanied by obvious systolic dysfunction, there were adjacent muscle tissues without injury. This injury is considered an acute severe GM contusion model [29, 30].

This study used 60 rabbits equally randomized into four groups: the normal group (*n* = 15, normal group), the contusion group with no treatment (*n* = 15, contusion group), the EA-treated experimental group (*n* = 15, EA group), and the recombinant human IGF-I- (rhIGF-I-) (PeproTech, NJ, USA) treated experimental group (*n* = 15, rhIGF-I group). Each group was then subdivided into three equal time point groups (days 7, 14, and 28 after contusion, *n* = 5 for each time point). All animals were reared in temperature (23 ± 1°C) and humidity (50 ± 5%) controlled rooms with 12-hour light-dark cycles. The animals had access to food and water ad libitum. All experimental procedures were approved by the Ethical Committee of the Academy of Medical Sciences and were conducted in accordance with international accepted principles for laboratory animal use and care.

### 2.2. Treatments

To keep the injury site dry and avoid infection, 25 g/L Entoiodine was applied topically once a day. The animals were treated with EA or rhIGF-I at 24 hours after contusion. EA was given for 15 minutes every other day with 0.4 mA (2 Hz). A needle of 0.25 mm in diameter and 25 mm in length (Zhongyan Taihe Medical Instruments Co. Ltd., Beijing, China) was used in this study. The main needle (as anode) was inserted into an acupoint area of the normal side ST36 (according to World Health Organization standards) with 15 mm. Then, the auxiliary needle (as cathode) was placed 5 mm away from the main needle. At the injured side, Ashi acupoints were located 10 mm from the proximal end (as anode) and the distal end (as cathode) at the contusion midpoint and handled with the needle liking ST36 in their acupoint areas. When all of the needles were placed, all of the electrodes were stimulated synchronously with identical parameters using Han's acupoint nerve stimulator (Han's 200E, Nanjing Jisheng Medical Co. Ltd., Jiangsu, China) (Figures 1(c)–1(f)). The rhIGF-I group was treated with 0.1 mg/mL rhIGF-I at a dose of 0.25 mL per rabbit. The rhIGF-I was injected into the GM once a week. The rabbits in the contusion group were allowed to recover naturally from the injury, which together with the normal and rhIGF-I groups received mock EA treatments (with the fixed position and time in the EA group but without EA treatments). The five rabbits from each subgroup were sacrificed on days 7, 14, or 28 after contusion.

### 2.3. The Evaluation of T-SOD and T-AOC Activities and MDA Level

On day 1 before contusion (as day 0) and days 1, 7, 14, and 28 after contusion blood was collected from the ear central artery and centrifuged. The blood was prepared in accordance with the requirements for each kit (Jiancheng Bioengineering, Nanjing, China). The blood was treated with the xanthine oxidase method for total superoxide dismutase (T-SOD) analysis, the iron reduction method for total antioxidant capacity (T-AOC), and the thiobarbituric acid method for malondialdehyde (MDA). The samples were assayed at 550 nm, 520 nm, and 532 nm using Varioskan Flash (Thermo, MA, USA). The spectrum scan and multifunction meter readings were performed with Plus SkanIt 2.4.3 RE software to record data. 

### 2.4. Local Microcirculation Detection

 On days 7, 14, and 28 after contusion, the animals were sedated with intravenous anesthesia, and the dorsal skin of their right hind limbs was opened surgically. Blunt dissection of the fascia tissues was performed, and the GM surfaces were scanned by a Laser Doppler Blood Perfusion Imaging (LDPI) (PeriScan PIM II type, PERIMED, Sweden). The scanning laser wavelength was 670 nm, and an NR scanning pattern with middle scanning accuracy was used. There was a 20 cm distance from the scanning head to the detected object, while the area of the scanning image was approximately 70 (width) × 70 (height) mm^2^, resulting in an image with a pixel size of 0.5 × 0.5 mm^2^. The time used for scanning one image was 45 seconds, and each area was scanned twice to achieve a mean value. The affiliated online LDPI with a 2.5 image analysis system was used to conduct body blood flow recording, analysis, processing, and storage. Blood flow values from the surface of the GM at the contusion site were then extracted. The blood flow measurement was calculated as perfusion units (PU) in a PERIMED instrument (PU= CMBC (the concentration of measuring the volume inside the blood cells) × V (the average velocity of blood cells)).

### 2.5. Histological Staining and Quantitative Histological Analysis

Histological analysis in the four groups at various time points after contusion was visualized by hematoxylin-eosin (HE) staining. Regenerative myofibers were distinguished by their centralized nuclei [31]. Nuclei with no discernible surrounding cytoplasm were discarded. The total number of regenerative myofibers within the contusion site was measured in 5 random fields of each sample by using a previously described protocol [32]. To measure the diameter of the regenerative myofibers, the minor axis diameters (the smallest diameter) were measured by Image-Pro Plus Image analysis software (IPP, Version 6.0, Media Cybernetics, USA). For each sample, six sections were randomly selected from the HE sections (original magnification, ×200) for image acquisition. This technique of measuring the smallest diameters of the centronucleated myofibers is a widely used method for evaluating muscle regeneration [33]. We followed a previously established protocol [34], and the diameters of centronucleated myofibers more than 10 *μ*m were consecutively measured in each GM. The prepared sections were observed by an investigator who was blinded to the experiment using light microscopy (ZEISS Scope. AI, Carl Zeiss, Germany).

### 2.6. Masson Staining and Immunohistochemistry (MHC, GDF-8, and p-Smad2/3)

GM samples were fixed in 4% formalin for 3 days before being embedded in paraffin. The blocks were cut into 5 *μ*m sections and were then dewaxed and rehydrated through a graded alcohol series. This protocol is often used for classical Masson staining and immunohistological staining. 

The classical Masson staining was used. Briefly, Masson's trichrome staining was performed to quantify collagen content along the zone of injury. This process stained the skeletal muscle fibers red. The collagen is stained slightly green, and the nuclei are stained black. For each sample, six sections were analyzed by IPP, and the percentage of the total collagen-positive area relative to the total cross-sectional area was calculated to estimate fibrosis formation (×200). 

Immunohistostaining: after retrieval, the sections were placed in 3% H_2_O_2_ for 10 minutes at room temperature (RT). After washing, the sections were blocked with blocking solution for 10 minutes at RT. Sections were incubated with the primary antibodies: MHC (1 : 50), GDF-8 (1 : 50), and p-Smad2/3 (1 : 50) rabbit polyclonal antibodies (Santa Cruz, CA, USA) overnight at 4°C. After washing, the sections were incubated at 37°C for 30 minutes with the following antibodies: polymerization HRP conjugated to anti-rabbit IgG (Wuhan Boster Biological Engineering Co. Ltd., China). After washing, the results were detected using a histochemical stain 3′-3′ diaminobenzidine (DAB, Wuhan boster biological engineering Co. Ltd., China) for 10 minutes at RT. Brown staining of the cytoplasm or nucleus was considered a positive result. When all of the staining was completed for each sample, six sections were randomly selected from immunohistochemical (×400) sections for image acquisition. Finally, the mean optical density (MOD) value was calculated by IPP. The prepared sections were observed by an investigator who was blinded to the experiment using light microscopy (ZEISS Scope. AI, Carl Zeiss, Germany).

### 2.7. Transmission Electron Microscopy (TEM) to Observe the Myofibril Ultrastructure

When the rabbits were sacrificed, a piece of GM was harvested (1 × 1 × 1 mm^3^). The injured GM tissues were fixed in 5% glutaraldehyde for 1–3 h, washed in buffer, and then fixed in 1% osmic acid with pH adjusted to 7.2–7.4 at 4°C. The tissues were dehydrated with an ethanol gradient and were then put in 100% acetone for 10 minutes. The tissues were then embedded in a mixture of 100% dehydrating agent and equivalent embedding medium (Epon 812) for 60 minutes, before they were finally placed in pure embedding medium overnight at 4°C.

The structures of myofibers were analyzed with an electron microscope (Hitachi Ltd., Japan). Tissue sections of 80 nm were prepared and stained with acetic acid uranium saturated aqueous solution, followed by lead citrate. The tissues were examined for morphology changes such as arrangement rules of myofibrils, position of the sarcomere, sarcomere light band (I line), and dark zones (A line). Any abnormal Z-Lines, skeletal muscle cell membrane, nucleus, mitochondria, sarcoplasmic reticulum, T small tube, satellite cell, and so forth were also observed.

### 2.8. Statistics

Data from this study are presented as the mean value ± standard deviation. Data from each time point were analyzed with SPSS 13.0 statistical software. The normal distribution was analyzed with single factor analysis of variance. The comparison between groups was performed using the LSD method. Outcomes were evaluated with double-sided inspection. A *P* < 0.05 was considered to have a significant difference.

## 3. Results

### 3.1. The Evaluation of Muscle Regeneration after Contusion

#### 3.1.1. A Quantitative Histological Analysis of Muscle Regeneration

**Figure 2:**
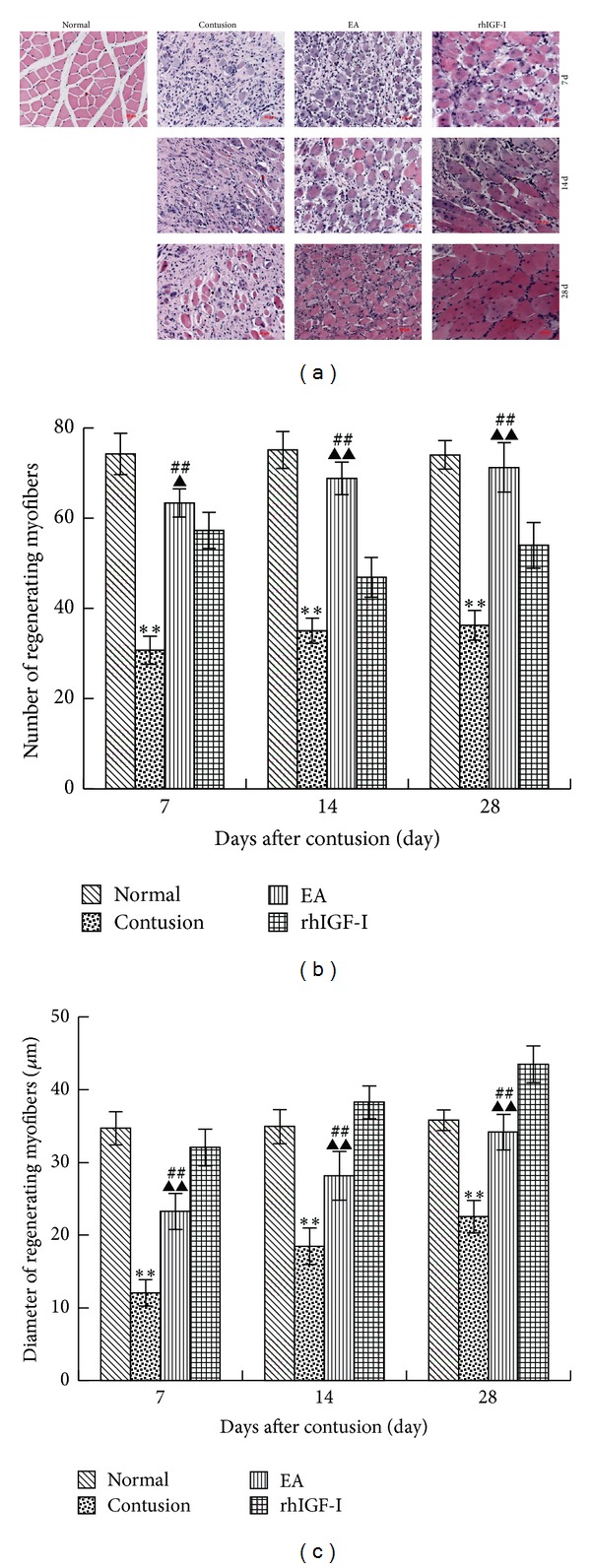
BBHistological analysis of the four groups at different time points after contusion was visualized by HE staining (×200) (a). A comparison of the number (b) and diameter (c) of regenerative myofibers in the different groups on days 7, 14, and 28 after contusion. The cytoplasm of regenerative myofibers was apparently basophilic, and more nuclei were located in the central area. In the contusion group, regenerative myofibers were surrounded by a mass of collagen fibers with small diameter. On day 7 in the EA group, the regeneration was stronger than that in the contusion group, but in the rhIGF-I group, the diameter of regenerative myofibers was wider than that in the normal group on day 28. In the contusion group, the number and diameter of regenerative myofibers were less than those of the normal group. The number in the EA group was markedly more than the contusion and rhIGF-I groups (*P* < 0.01 or *P* < 0.05). The diameter of fibers in the EA group was wider than that in the contusion group (*P* < 0.01) but narrower than that in the rhIGF-I group (*P* < 0.01). Contusion versus normal, ^★^ *P* < 0.05, ^★★^ *P* < 0.01; EA versus contusion, ^#^ *P* < 0.05, ^##^ *P* < 0.01; and EA versus rhIGF-I, ^▲^ *P* < 0.05, ^▲▲^ *P* < 0.01 (*n* = 5).

We observed that the injured gap was initially filled with a large hematoma and proliferating granulation tissue and that connective scar tissue developed in the injured site within 14 days after contusion. In this study, after contusion regenerative myofibers showed basophilic cytoplasm, and more trachychromatic nuclei were gathered in the central area (Figure 2(a)).

As the contusion healed, the centronucleated regenerative myofibers were replaced by myofibers with a larger diameter and nuclei located at the periphery. In the contusion group, regenerative myofibers were surrounded by a large number of collagen fibers. The number and diameter of collagen fibers were significantly lower than those of the normal group (*P* < 0.01). After the EA treatment, myofiber regeneration occurred and this continued until day 28. The number and diameter of regenerating myofibers were dramatically increased compared to those of the contusion group on day 7 (*P* < 0.05 or *P* < 0.01). However, there were no significant differences observed between the normal and EA groups on day 28 (*P* > 0.05). Additionally, regenerative myofibers were also found in the rhIGF-I group, and their diameter was significantly greater than that of the EA group (*P* < 0.01). However, the number of fibers was lower than that of the EA group (*P* < 0.05 or *P* < 0.01) (Figures 2(b)-2(c)).

#### 3.1.2. The Expression of MHC in Different Groups

MHC is an important structure of myosin filaments in sarcomeres and is responsible for maintaining the structural integrity of myofibers. In the processes of skeletal muscle development and regeneration, MHC in the cytoplasm of the muscular tube marks myofiber regeneration [24]. Therefore, the expression intensity of MHC in the cytoplasm has been used to evaluate myofiber regeneration [25, 26].

**Figure 3:**
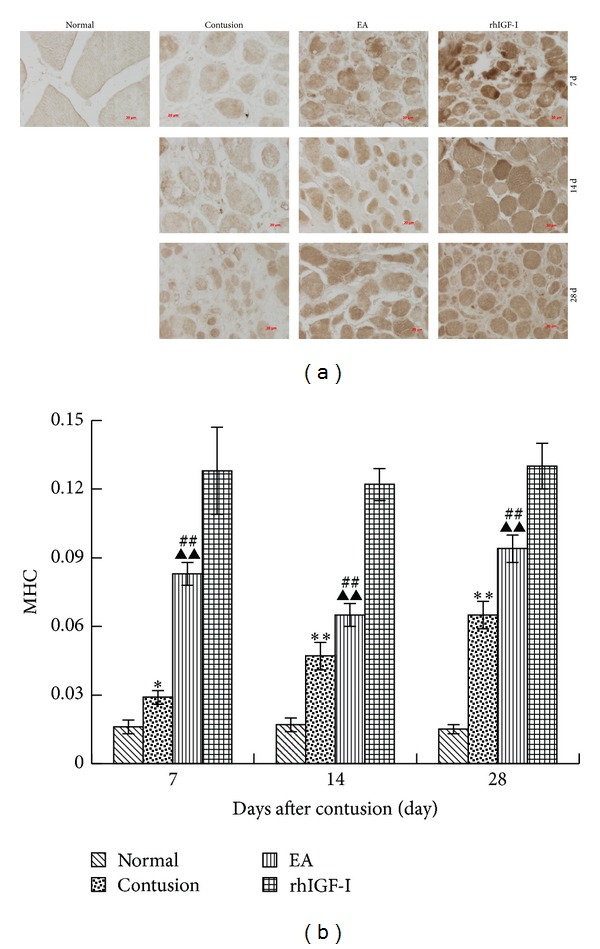
DAB staining of MHC on days 7, 14, and 28 after contusion (×400) (a). The comparison of the MHC levels on days 7, 14, and 28 post-contusion (b). Positive expression of MHC in the cytoplasm of regenerative myofibers stains brown. MHC staining was weak in the normal group and weaker in the contusion group. MHC staining in the EA and rhIGF-I groups was enhanced, particularly in the rhIGF-I group. In the contusion group, the MHC level was significantly higher than that in the normal group (*P* < 0.01 or *P* < 0.05), while the EA group was dramatically higher than the contusion group (*P* < 0.01) but lower than the rhIGF-I group (*P* < 0.01). Contusion versus normal, ^★^ *P* < 0.05, ^★★^ *P* < 0.01; EA versus contusion, ^#^ *P* < 0.05, ^##^ *P* < 0.01; and EA versus rhIGF-I, ^▲^ *P* < 0.05, ^▲▲^ *P* < 0.01 (*n* = 5).

We found that MHC expression in normal myofibers rarely existed and that the MOD value of MHC in the contusion group was also markedly higher than the normal group on days 7, 14, and 28 after contusion (*P* < 0.01 or *P* < 0.05). The MOD value of MHC in EA group was significantly higher than that the contusion group (*P* < 0.01) but lower than that the rhIGF-I group (*P* < 0.01) (Figure 3).

#### 3.1.3. Ultrastructure Changes of Skeletal Muscles after Injury in Different Groups

**Figure 4:**
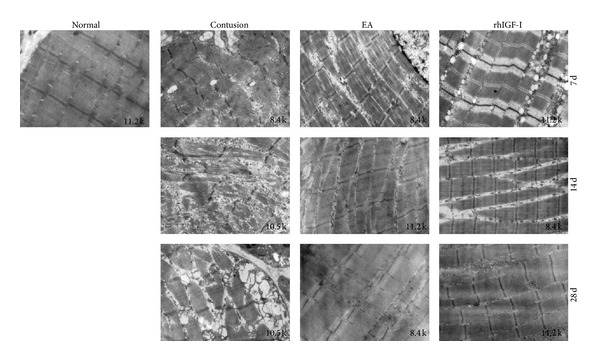
The ultrastructure of GM on days 7 and 14, and after contusion. On day 7 post-contusion, the structure of myofibrils and sarcomeres in the contusion groups was disordered. In the contusion group, the Z-Line was vague and streaming. The swelling of the mitochondria and sarcoplasmic reticulum was uniform in the rhIGF-I group and the number of both organelles increased. On day 14 after contusion, myofibrils were small and the diameter was different in the contusion group. In the EA and rhIGF-I groups, the arrangement between myofibrils appeared like the connection of joints: the Z-Line was clear and regular and the diameter of myofibrils was uneven. On day 28 post-contusion, the Z-Lines disappeared in some myofibrils in the contusion group and some were atrophic. The mitochondria gathered under the sarcolemma like a large bubble. The structures of the myofibril, sarcomere, and Z-Line were regular and mitochondria returned to normal in both the EA and rhIGF-I groups.

TEM shows ultrastructural changes of myofibers, especially sarcomeres and mitochondrial changes. Sarcomere structure reflects the function of myofibers, and changes of mitochondria show the state of myofibers. In the normal group, GM sarcomeres had a normal structure, which was clear and regular, and the myofilaments were arranged in an orderly fashion. There was a small amount of mitochondria between myofibrils, and the sarcoplasmic reticulum distribution was regular with an integrated muscle membrane. The number of satellite cells located in the basement membrane was reduced, and they had a spindle shape with a small amount of cytoplasm. On day 7 after contusion, the structure of myofibrils and sarcomeres in the injured groups was disordered and the Z-Line, mitochondria, and sarcoplasmic reticulum were abnormal. The satellite cells were activated after injury. In the contusion group, the Z-Line was vague and had obvious streaming. The swelling of the mitochondria and sarcoplasmic reticulum was uniform in the rhIGF-I group and both were increased in number. On day 14 after contusion, the number of swelling mitochondria in the injured groups decreased. In the contusion group, myofibrils were small and the diameters were different. In the EA and rhIGF-I groups, the arrangement between myofibrils appeared like a connection of joints, with the Z-Line being clear and regular. However, the diameter of myofibrils was uneven. On day 28 after contusion, Z-Lines disappeared in some myofibrils in the contusion group and some were atrophic with mitochondria gathered under the muscle membrane like a bubble. The structures of the myofibril, sarcomere, and Z-Line were regular, and mitochondria returned to normal in the EA and rhIGF-I groups (Figure 4).

### 3.2. EA Influences the Microcirculation of the GM and Antioxidant Ability

#### 3.2.1. Microcirculation Changes of GM after Injury

LDPI can detect microcirculation status after a direct stress reaction and has been widely used in clinical diagnosis, evaluation, and research associated with microcirculation such as acupuncture [35] and skeletal muscle microcirculation [36]. We measured the number of perfusion units in this study: PU = CMBC (the concentration of measuring the volume inside the blood cells) × V (the average velocity of blood cells).

**Figure 5:**
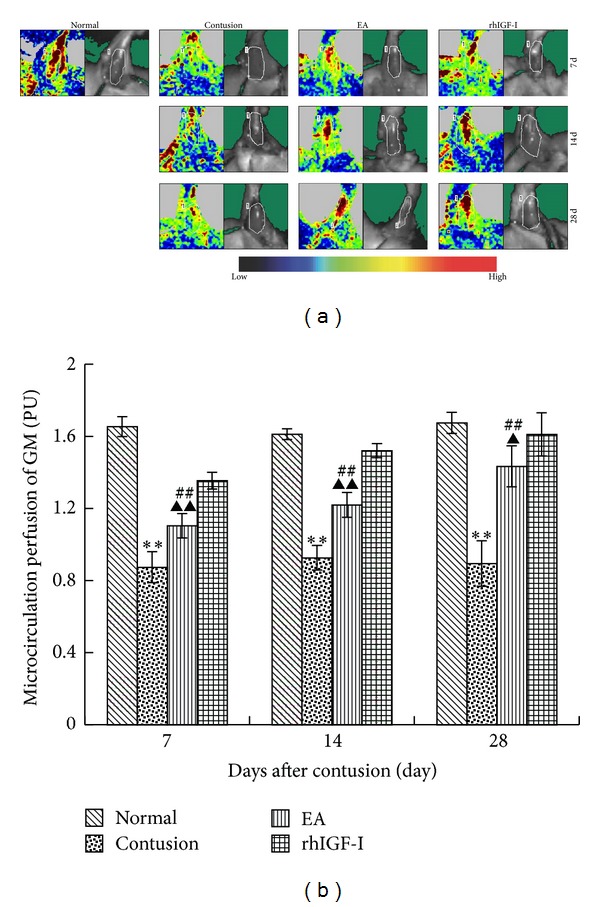
Repeated use of LDPI at the contusion site in different groups after contusion (a). Comparisons of the microcirculation perfusion of GM on days 7, 14, and 28 after contusion (b). In the contusion group, the contusion area was mainly blue and green, with only a small amount of yellow and red. On day 7 in the EA and rhIGF-I groups the images were mostly green, yellow, and red. The red area was expanded on days 14 and 28. The microcirculation perfusion in the contusion group was markedly lower than that in the normal group (*P* < 0.01). The EA group was apparently more than the contusion group (*P* < 0.01) but significantly lower than the rhIGF-I group (*P* < 0.05 or *P* < 0.01). Contusion versus normal, ^★^ *P* < 0.05, ^★★^ *P* < 0.01; EA versus contusion, ^#^ *P* < 0.05, ^##^ *P* < 0.01; and EA versus rhIGF- I, ^▲^ *P* < 0.05, ^▲▲^ *P* < 0.01 (*n* = 5).

The PU values of the contusion groups were significantly lower (*P* < 0.01) than those of the normal group on days 7, 14, and 28 after contusion. The EA group was apparently more than the contusion group (*P* < 0.01) but significantly lower than the rhIGF-I group (*P* < 0.05 or *P* < 0.01). As the process of repair continued, the blood perfusion in the EA and rhIGF-I groups increased (Figure 5).

#### 3.2.2. The Expression of T-SOD, T-AOC, and MDA in Different Groups

**Figure 6:**
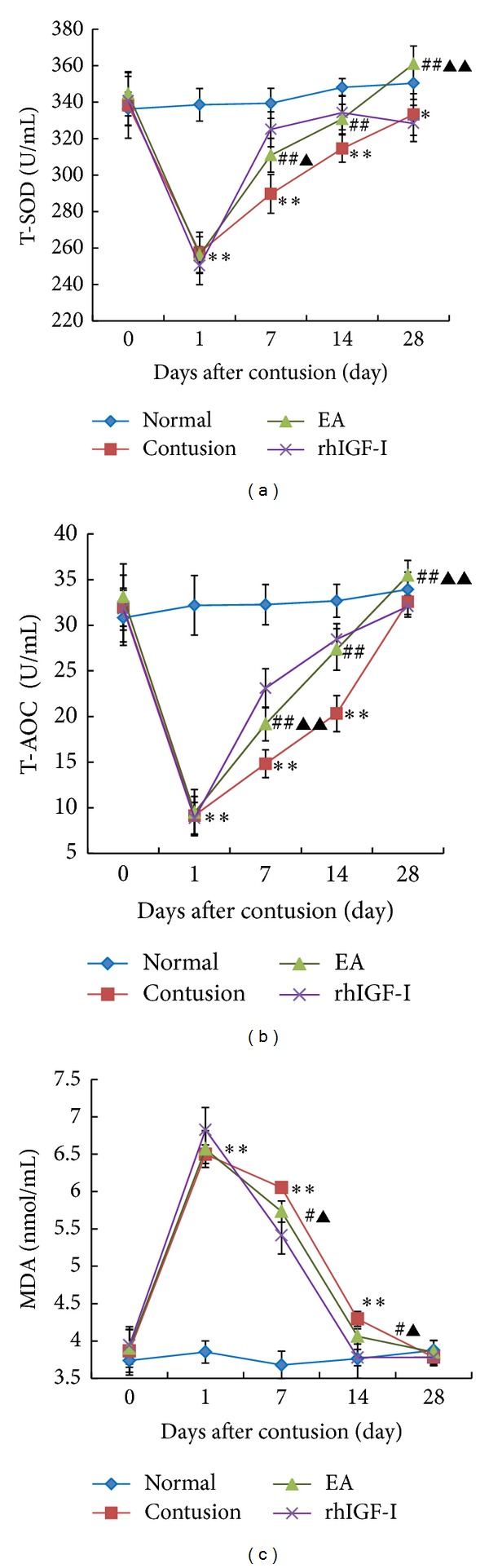
Comparisons of serum T-SOD (a), T-AOC (b), and MDA (c) before 1 day as 0 d and after 1d, 7d, 14d, and 28d contusion. After contusion, the activities of the T-SOD and T-AOC dramatically decreased, while the level of MDA sharply increased. The serum levels gradually returned to normal. On days 7, 14, and 28 after contusion, T-SOD and T-AOC activities in the EA group were significantly higher than those of the contusion group (*P* < 0.01) but lower than those of the rhIGF-I group (*P* < 0.01 or *P* < 0.05) on day 7. On day 28, the EA group was significantly higher compared to the rhIGF-I group (*P* < 0.01). On days 7 and 14 after contusion, MDA level in the EA group was significantly lower than that in the contusion group (*P* < 0.05) but higher than that in the rhIGFI group (*P* < 0.05). Contusion versus normal, ^★^ *P* < 0.05, ^★★^ *P* < 0.01; EA versus contusion, ^#^ *P* < 0.05, ^##^ *P* < 0.01; and EA versus rhIGF-I, ^▲^ *P* < 0.05, ^▲▲^ *P* < 0.01 (*n* = 5).

To investigate the protective effects of EA in oxidative stress responses after contusion, we examined the activities of serum T-SOD, T-AOC, and MDA. On day 1 after contusion, the activities of T-SOD and T-AOC decreased significantly and were lower than that of the normal group (*P* < 0.01). However, the MDA group showed the opposite results (Figure 6). 

During muscle injury repair, T-SOD and T-AOC activities in each contusion group increased gradually and returned to normal by 28 days. Conversely, serum MDA level gradually decreased to normal levels. On days 7, 14, and 28 after contusion, T-SOD and T-AOC activities in the EA group were significantly higher than those of the contusion group (*P* < 0.01) but lower than those of the rhIGF-I group (*P* < 0.01 or *P* < 0.05) on day 7. There were few differences between the EA and rhIGF-I groups on day 14 (*P* > 0.05). On day 28, the EA group was significantly higher compared to the rhIGF-I group (*P* < 0.01). On days 7 and 14 after contusion, MDA level in the EA group was significantly lower than that in the contusion group (*P* < 0.05) but higher than that in the rhIGF-I group (*P* < 0.05) (Figure 6).

### 3.3. EA Influences the Density of Collagen Fibers, GDF-8, and p-Smad2/3

#### 3.3.1. Density of Collagen Fibers in Different Groups

**Figure 7:**
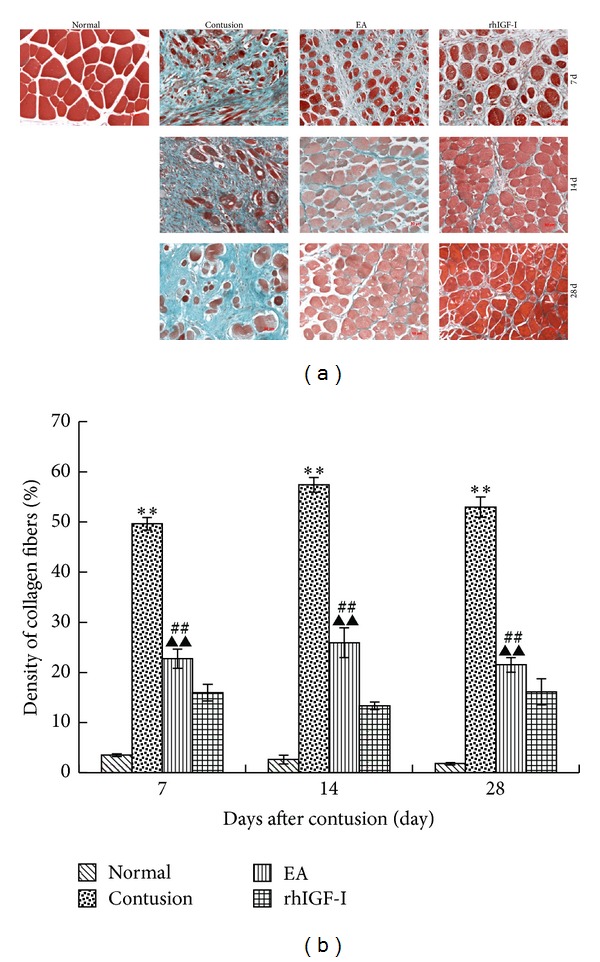
Collagen fibers distribution on days 7, 14, and 28 after contusion among different groups (×200) (a). By using Masson's trichrome staining, we confirmed that a large amount of collagen was deposited in the contusion site. The collagen levels were significantly decreased in both the EA and rhIGF-I groups. Masson's trichrome staining showed myofibers (red) and collagen (green) among different groups. The density of collagen (b) in the contusion was higher than that in the normal group (*P* < 0.01). The collagen in the EA group was lower than that in the contusion group (*P* < 0.01) but markedly higher than the rhIGF-I group (*P* < 0.01). Contusion versus normal, ^★^ *P* < 0.05, ^★★^ *P* < 0.01; and EA versus contusion, ^#^ *P* < 0.05, ^##^ *P* < 0.01; EA versus rhIGF-I, ^▲^ *P* < 0.05, ^▲▲^ *P* < 0.01 (*n* = 5).

We examined the extent of fibrosis of the local extracellular matrix (ECM) after contusion. Masson trichrome staining was performed to detect the ratio of the fibrotic area to the total cross-sectional area of the muscle. There were small green stained collagen fibers around normal myofibers. During the repair process, the green collagen fibers around red dyed myofibers filled the area outside muscle cells. On day 7 after contusion, collagen fibers were deposited around the myofibers. On days 7, 14, and 28 after contusion, the density of collagen fibers in the contusion group was significantly higher than that in the normal and EA groups (*P* < 0.01). Additionally, the EA group had more fibers than the rhIGF-I group (*P* < 0.01) (Figure 7).

#### 3.3.2. The Expression of GDF-8 and p-Smad2/3 in Different Groups

**Figure 8:**
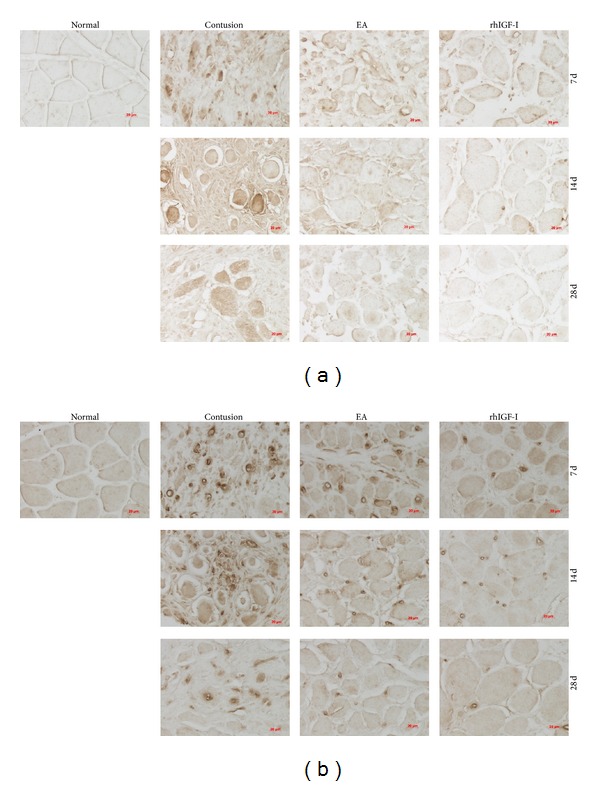
GDF-8 (a) and p-Smad2/3 (b) staining on days 7, 14, and 28 after contusion (×400). GDF-8 in the cytoplasm and p-Smad2/3 in the nucleus appear brown. The staining was weak in the normal group and obvious in the contusion group. The staining was weaker in the EA and rhIGF-I groups, especially on day 28.

**Figure 9:**
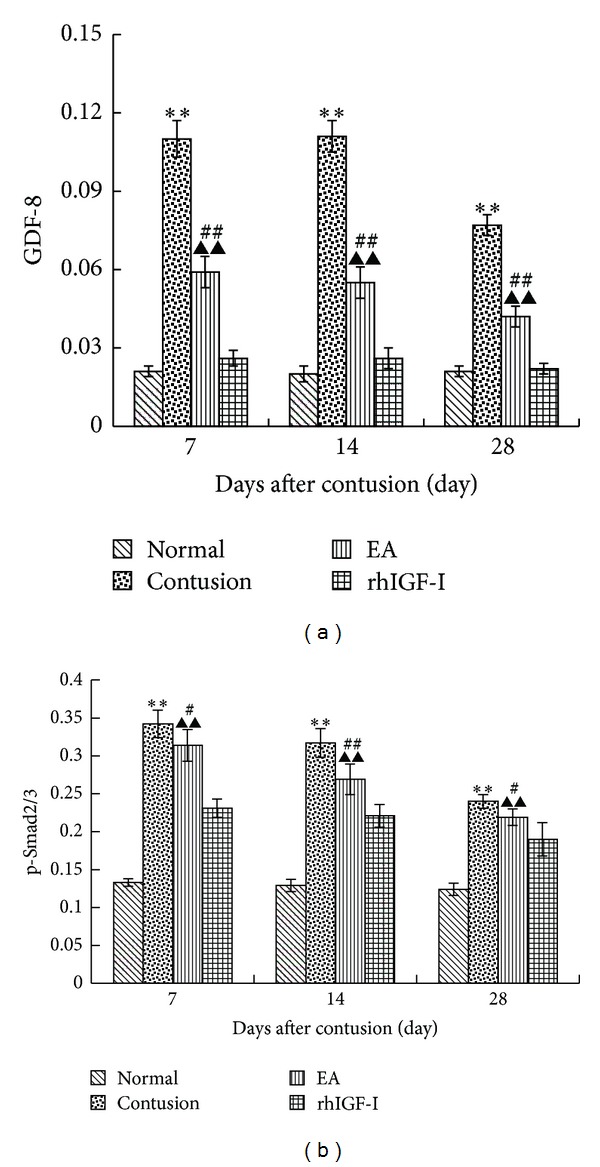
Comparisons of GDF-8 (a) and p-Smad2/3 (b) expression in different groups on days 7, 14, and 28 after contusion. The density of collagen and expression of GDF-8 and p-Smad2/3 in the contusion group were higher than those in the normal group (*P* < 0.01). The EA group had less expression than the contusion group (*P* < 0.05 or *P* < 0.01) but markedly higher expression than the rhIGF-I group (*P* < 0.01). Contusion versus normal, ^★^ *P* < 0.05, ^★★^ *P* < 0.01; and EA versus contusion, ^#^ *P* < 0.05, ^##^ *P* < 0.01; EA versus rhIGF- I, ^▲^ *P* < 0.05, ^▲▲^ *P* < 0.01 (*n* = 5).

To analyze the mechanism how EA inhibits excessive ECM fibrosis, we investigated the GDF-8/p-Smad2/3 signaling pathway. We found that on days 7, 14, and 28 after contusion the protein expression levels of GDF-8 and p-Smad2/3 in the contusion group were significantly higher than those in the normal group (*P* < 0.01). The EA group was significantly higher than the contusion and rhIGF-I groups (*P* < 0.01). This trend was consistent with the changes in the density of collagen fibers (Figures 8 and 9). 

## 4. Discussion

Acupuncture has a long history of use in China and throughout Asia. The curative effects of acupuncture are not well known, which limits its use globally. Therefore, it is very important to define the benefits of acupuncture. As one of the modern methods of acupuncture, EA uses the acupuncture needle as an electrode to apply low-frequency stimulation. Evidence suggests that EA is safe and effective in a wide variety of diseases such as polycystic ovary syndrome [37], dysmenorrhea [38], and autism spectrum disorder [39].

Skeletal muscle is crucial for structural support, movement, and function. One of the most common causes of muscle contusion is the impact of a nonpenetrating object [40]. Once the contusion injury occurs, the process of healing is activated and myofibers have the ability to regenerate through the activation of satellite cells [40]. However, fibrosis formation can ultimately impair the muscle healing process due to the accumulation of excessive collagen. Fibrosis can even become a self-perpetuating process, and excessive fibrosis inhibits regeneration of myofibers [41].

Based on previous studies, we investigated the mechanism of EA effects on promoting skeletal muscle regeneration and inhibiting excessive fibrosis after contusion. This study observed the healing process of rabbit GM after contusion and showed that EA treatment could significantly increase the number and diameter of regenerative myofibers and improve MHC level. Additionally, the arrangement of myofilaments within the myofibril and Z-Line distribution appeared to normalize when analyzed by TEM. The contusion group had myofibril fracture and atrophy. This result indicates that EA not only could promote myofiber regeneration but also improve the restoration of myofibril and sarcomere structure. 

The natural healing area contained regions of fibrosis with a large number of collagen fibers tightly surrounding the myofibers and evidence of myofiber atrophy. The EA treatment could effectively relieve fibrosis and reduce myofiber atrophy. The surrounding tissues and blood vessels were also damaged so the supply with nutrients to nerves and myofibers was obstructed. This obstruction caused secondary damage [42], and blood circulation disturbances are an important cause of enhanced ECM fibrosis and reduced myofiber regeneration. Thus, proper blood perfusion is essential to heal injured muscle [43]. Previous studies reported that electroacupuncturing the ST36 [44] or Ashi acupoints [45] improved blood flow. We assessed microcirculation changes of GM after injury and found that the EA group was more effectively repaired than the contusion group. Additionally, there were significant differences between the EA and contusion groups. Our data demonstrated that electro-acupuncturing the ST36 and Ashi acupoints improved the velocity of blood flow and promoted the absorption of hematomas. These improvements may be related to the overall effect. 

Contusion can damage the integrity of plasma membranes and the basilar membrane of the skeletal muscle fibers. The damage causes extracellular calcium ion flux and local hematoma formation, activation of neutrophils and macrophages, and induction of “respiration burst.” The respiration burst results in the formation of superoxygen ions. The oxygen free radicals damage large cellular molecules such as lipids, proteins, and DNA, ultimately leading to cell death and causing further damage of skeletal muscle [46]. Therefore, antioxidants could effectively reduce tissue fibrosis [47] and be beneficial to myofiber structure reconstruction [48] by preventing myofiber atrophy [49].

Electro-acupuncturing the ST36 acupoint protects the body through antioxidation. EA protects the hypothalamus, the liver, red blood cells [50], and the substantia nigra striatum [51], and so forth. T-AOC consists of enzymatic and nonenzymatic antioxidant defense systems and reflects the body's ability to regulate antioxidants and scavenge free radicals. Low levels of T-AOC can reduce antioxidant enzyme synthesis in the body or utilize the nonenzymatic antioxidants to control excessive reactive oxygen species (ROS) generated over the oxidation/antioxidation equilibrium. EA increased the T-AOC level in injured tissues, playing a protective role [52]. T-SOD is the main antioxidant enzyme within skeletal muscle, and it can remove free radicals and peroxides produced by tissues and cells metabolism, protecting cells against oxidative stress damage [53]. In this study, on day 1 after contusion, the activities of serum T-AOC and T-SOD decreased significantly and then increased gradually. The decreased level of serum T-SOD in our experiment might be due to the inhibition or oxidative inactivation of enzyme proteins in the serum caused by excess generation of ROS [54]. The activities of serum T-AOC and T-SOD in the EA group returned to normal gradually and were higher than those of the contusion group. The enhancement of T-AOC may increase the resistance to oxidative cellular injury or facilitate the biosynthesis in cells which receive sublethal injury [55]. These possibilities are consistent with the results of our experiments. EA could also reduce undesired oxidation by infiltrating cells and/or could facilitate tissue repair. MDA is one of the major secondary oxidation products derived from polyunsaturated fatty acids [56], so the detection of MDA level can reflect the degree of lipid peroxidation in the body and the degree of cell injury. We found that the increase of MDA level could directly reflect oxidative damage of myofibers on day 1 after contusion. After EA treatment, MDA level decreased significantly. This result shows that the removal of oxygen free radicals increased because EA improved the circulation. The improved circulation protects the cell membrane from the attack of oxygen free radicals and reduces the generation of lipid peroxidation products.

Oxidative stress causes a catastrophic cycle of mitochondrial DNA (mtDNA) damage and functional decline. This creates further reactive oxygen species generation and cellular injury [57]. This study confirmed that EA improved mitochondrial function in injured tissues [58], and electro-acupuncturing the ST36 acupoint [59] relieved the swelling of mitochondria. On day 28, the contusion group showed mitochondrial changes accompanied by myofibril fracture and atrophy. However, the EA or rhIGF-I treatment groups did not show a similar phenomenon. This phenomenon might be related to the GM microcirculation perfusion inadequacy because the contusion group had mitochondria swelling. Therefore, EA activated blood circulation and gradually improved microcirculation in the skeletal muscle after contusion. The improved circulation increased oxygen radical scavenging ability and improved the antioxidant ability. Additionally, increased circulation could also help improve injured tissue energy supplies, reduce oxidative stress damage, relieve cellular infiltration, protect sarcomere structure, and eventually reduce fibrosis. These properties are advantageous to myofibril regeneration.

Fibroblasts were activated after injury of skeletal muscle and secreted collagen fibers for several weeks [60]. Collagen fibers in the early phase of wound healing have a positive effect and can increase the tensile strength of the wound and allow the wound on both ends of the fibers to attach to the skeleton [61]. As the repair is completed, the earliest granulation tissues are replaced by scar tissue (mainly I type collagen) and remain constant for a long time [62]. The scar tissue is an obstacle to new fibers and inhibits skeletal muscle regeneration [11]. Previous studies have shown that excessive fibrosis of ECM was an important factor that impaired myofiber regeneration [33]. Therefore, we speculated that EA could promote myofiber regeneration by inhibiting excessive fibrosis. Thorsteinsdottir et al. [63] indicated that degrading ECM and reducing fibrosis were advantageous to satellite cell migration and differentiation and promoted skeletal muscle regeneration. In organ fibrosis research, electro-acupuncturing the ST36 alleviated renal failure-induced glomerulosclerosis, tubulointerstitial fibrosis [64], and carbon tetrachloride-induced hepatic fibrosis [65]. To investigate whether EA can reduce ECM fibrosis of skeletal muscle after contusion, this study observed the fibrosis formation process in the damaged region repeatedly on days 7, 14, and 28. The results showed that fibrosis in the contusion group was obvious, but the EA group inhibited collagen fiber deposition from the 7th day and had significantly reduced overall fibrosis. 

To investigate the mechanism of how EA inhibits ECM fibrosis, we analyzed effects of EA on the GDF-8/p-Smad2/3 signaling pathway. GDF-8 as one member of the TGF-*β* superfamily [66] is involved in accelerating the deposition of ECM by increasing the synthesis of ECM proteins and inhibiting ECM degradation [40]. It is also a negative regulator of skeletal muscle growth [66], and its mRNA and protein are highly expressed in degenerating skeletal muscle and connective tissues [67]. GDF-8 also inhibits the self-renewal of muscle satellite cells [68] by delaying myogenin expression [69] and enhancing fibrosis through the activation of the typical TGF-*β*/Smad signaling pathway [70]. p-Smad2/3 are profibrotic factors linked to GDF-8 in the nucleus where it induces the process of deposition of ECM fibrosis [71]. GDF-8 also has a regulatory role in the proliferation of dystrophic muscle fibroblasts [41] and myoblast differentiation [72]. TGF-*β* is required to activate the Smad2/3 signal transduction pathway because the effect could be blocked using a TGF-*β* neutralizing antibody [73]. On days 7, 14, and 28 after contusion, EA could significantly inhibit GDF-8 and p-Smad2/3 protein expression levels. EA not only reduced fibrosis by inhibiting the TGF-*β*/Smad signaling pathway but also improved myofiber regeneration by inhibiting GDF-8. 

 In conclusion, these results suggest that EA promotes skeletal muscle regeneration and inhibits excessive fibrosis after contusion, and the mechanism may be associated with several factors. These factors include its effect on improving local microcirculation perfusion with promoting blood circulation to remove blood stasis. Additionally, EA increased the ability to resist oxidative stress, which protected skeletal muscle from secondary injury. EA also inhibited ECM fibrosis and provided a good environment for skeletal muscle regeneration by enhancing collagen degradation ability and inhibiting the TGF-*β*/Smad signaling pathway. And some functional assessments would be added in the further design to reveal the significance of EA treatments on muscle strength and electrophysiology after contusion.
